# A Monte Carlo model of the Dingo thermal neutron imaging beamline

**DOI:** 10.1038/s41598-023-44035-4

**Published:** 2023-10-13

**Authors:** Klaudiusz Jakubowski, Andrew Chacon, Linh T. Tran, Attila Stopic, Ulf Garbe, Joseph Bevitt, Scott Olsen, Daniel R. Franklin, Anatoly Rosenfeld, Susanna Guatelli, Mitra Safavi-Naeini

**Affiliations:** 1 Centre for Medical Radiation Physics, University of Wollongong, Wollongong, NSW 2522 Australia; 2 Australian Nuclear Science and Technology Organisation, Sydney, NSW 2234 Australia; 3 School of Electrical and Data Engineering, University of Technology Sydney, Sydney, NSW 2007 Australia

**Keywords:** Design, synthesis and processing, Experimental particle physics, Statistical physics, Experimental nuclear physics

## Abstract

In this study, we present a validated Geant4 Monte Carlo simulation model of the Dingo thermal neutron imaging beamline at the Australian Centre for Neutron Scattering. The model, constructed using CAD drawings of the entire beam transport path and shielding structures, is designed to precisely predict the in-beam neutron field at the position at the sample irradiation stage. The model’s performance was assessed by comparing simulation results to various experimental measurements, including planar thermal neutron distribution obtained in-beam using gold foil activation and $^{10}$B$_{4}$C-coated microdosimeters and the out-of-beam neutron spectra measured with Bonner spheres. The simulation results demonstrated that the predicted neutron fluence at the field’s centre is within 8.1% and 2.1% of the gold foil and $^{10}$B$_{4}$C-coated microdosimeter measurements, respectively. The logarithms of the ratios of average simulated to experimental fluences in the thermal (E$_{th}<$ 0.414 eV), epithermal (0.414 eV < E$_{epi}<$ 11.7 keV) and fast (E$_{fast}>$ 11.7 keV) spectral regions were approximately − 0.03 to + 0.1, − 0.2 to + 0.15, and − 0.4 to + 0.2, respectively. Furthermore, the predicted thermal, epithermal and fast neutron components in-beam at the sample stage position constituted approximately 18%, 64% and 18% of the total neutron fluence.

## Introduction

ANSTO’s Open-pool Australian Lightwater (OPAL) Nuclear Reactor in Sydney, Australia, uses low-enriched uranium fuel ($<20\%$ $^{235}$U) and provides neutron beams to 15 instruments, including diffractometers, reflectometers, spectrometers and neutron scattering instruments. A key beamline at OPAL is the Australian Centre for Neutron Scattering (ACNS) Dingo high-flux thermal neutron beamline. It is predominantly utilised for neutron radiography and tomography, in applications such as the study of the composition of fossils and ancient artefacts, quality control of pyrotechnic devices, water damage in aircraft components and flow of fuel and lubricants in aerospace or automotive engines^1^.

Thermal neutron sources are essential for research on neutron-based radiation therapy modalities such as Neutron Capture Therapy (NCT) and Neutron Capture Enhanced Particle Therapy (NCEPT), both leveraging neutron capture events in a tumour-targeting agent with high thermal neutron capture cross-section (such as $^{10}$B or $^{157}$Gd)^2–5^. Ongoing research programs focus on new neutron capture agent discovery, improved clinical protocols and dosimetric and image-based treatment verification / quality assurance methods^6–9^.

Dingo is presently the only thermal neutron imaging beamline available in Australia, and as such, is of critical importance to Australian researchers in a wide range of disciplines. Due to the high demand for access to Dingo, it is imperative that experiments on Dingo be optimised to make efficient use of time allocation. Analytical optimisation of experimental design is difficult due to the complexity of the beam and its surrounding environment—in particular, the neutron beam spectrum, field shape, the presence of a gamma component to the field, and the effects of the composition of the room in which the beam emerges. A more practical approach would be to use a Monte Carlo simulation model of the beamline, which could then be used to evaluate a proposed experimental configuration. This “digital twin” would enable the parameters of the experiment to be explored in the simulation to determine the best beamline configuration and exposure intervals to maximise the likelihood of obtaining the desired results, to validate proposed dosimetric and spectrum measurement sensors, instruments and protocols and to estimate post-experiment activity levels in the experimental materials. It would also enable probing phenomena that are challenging or impossible to observe otherwise, such as prompt gamma emission or the decay of very short half-life isotopes. Such a model would therefore be a valuable tool for both users and beamline scientists.

This is the first Monte Carlo model of the Dingo beamline and to our best knowledge, the first high-resolution CAD-based Monte Carlo model of a reactor-based thermal neutron beamline in general. However, similar models have been created in the past for other neutron beamlines, for instance the TRIUMF accelerator-based thermal neutron facility in Canada, modelled in MCPNX^10^; the cold neutron facility (ICON) at the Swiss Spallation Neutron Source (SINQ), also modelled in MCPNX^11^, neutron imaging facility at TRIGA reactor in Morocco, modelled using Geant4^12^ and the high energy neutron irradiation facility at the China Spallation Neutron Source, modelled in MCNPX^13^. These models were designed for a range of different purposes, such as the characterisation of the high-energy neutron field, coupling of the MCNPX and McStas packages, neutron beamline design or the generation of preliminary data for the physical experiments.

The in-beam neutron spectrum at the Dingo beamline has not, as yet, been measured in detail; out-of-beam spectral measurements performed with Bonner spheres revealed that the neutron field in this region comprises a broad spectrum of thermal, epithermal and fast neutrons. Neutron spectral characterisation is usually performed with a set of Bonner spheres to cover a broad energy range of up to 20 GeV^14,15^, which can be extended by adding high-*Z* materials to the polyethylene moderator^16,17^. Bonner spheres offer an almost isotropic response across the entire energy range, but feature poor energy resolution^18^. Direct in-beam measurements with a LiF or $^3$He detector in active mode are challenging due to the high flux of the beam, which leads to a pile-up effect. This can be ameliorated by using a diamond detector or inserting activation materials into the polyethylene moderator^19,20^. An alternative approach to spectroscopy is Neutron Activation Analysis (NAA), which is a passive technique based on the relative quantity of different activation products detected following irradiation of a range of isotopically pure materials in the beam^21^. Unfortunately, NAA provides a non-unique solution. Both Bonner sphere and neutron activation methods rely on an unfolding (deconvolution optimisation) algorithm, which depends on a priori information, called the initial or guess spectrum^22–30^. This guess spectrum can be obtained using Monte Carlo simulation models, for example using Geant4, MCNPX or PHITS^31,32^.

In this work, we aim to develop an experimentally-validated Monte Carlo simulation model of the ACNS Dingo beamline. In-beam validation of the predicted transaxial distribution of thermal and epithermal neutrons at the sample stage position is performed using gold foil activation and Silicon on Insulator (SOI) microdosimeters covered with $^{10}$B$_{4}$C, while Bonner spheres are used to measure the neutron spectrum out-of-beam (the high neutron flux means that Bonner sphere spectroscopy cannot be used in-beam due to detector saturation). Gold is chosen for NAA since it has a high thermal neutron capture cross-section, and activation of natural high-purity $^{197}$Au results in the production of $^{198}$Au, which decays with a half-life of 2.7 days with a single emission wavelength of 411 keV. The $^{10}$B$_{4}$C-coated microdosimeter is another effective thermal/epithermal neutron relative detector due to the high neutron capture cross-section of $^{10}$B and high-LET secondary products from the $^{10}$B(n,$\alpha$)$^7$Li reaction, due to which it generates a strong signal with minimal noise^33,34^. First, CAD drawings of the Dingo beamline were imported into Geant4 via the CADMesh single-header interface^35^. The imported model extends from the reactor pinhole to the beam stop and includes part of the primary wall surrounding the reactor core, shielding walls and roof, the complete beam transportation system, relevant sample room components as well as spectroscopic and dosimetric instrumentation. Secondly, the thermal and epithermal neutron components’ spatial distribution scored in-beam was compared to previous neutron fluence measurements with gold activation foils and $^{10}$B$_{4}$C-coated microdosimeters. Then, out-of-beam neutron spectra were simulated and compared to the Bonner sphere measurements in eight locations, parallel to the central axis of the beam. Finally, the in-beam neutron spectrum at the sample stage position was predicted through Geant4 simulations. The output of this model is used to generate a validated phase-space file, which contains information about each particle crossing a phase-space plane, including its type, position and momentum. The resulting phase space file and energy spectrum can then be provided to users on request so that they can perform simulations without the need to load or model the complex geometry of the beamline themselves.

The methodology implemented to import the model of the ACNS Dingo beamline into Geant4 is briefly explained in “Simulation configuration” section. “[Sec Sec4]” and “Simulation of the neutron field spatial distribution at the sample stage” sections describe the experimental neutron fluence measurements with gold activation foils and SOI microdosimeters, and the simulations of the planar neutron field distribution at the sample stage, respectively. The out-of-beam Bonner sphere measurements are discussed in “Absolute out-of-beam Bonner spheres spectroscopy” section, while the simulation of the out-of-beam neutron spectra and the method to predict the in-beam neutron spectrum in “Simulation of absolute out-of-beam neutron spectra” section. The simulation results and experimental validation are demonstrated in “Results” section and discussed in “Discussion” section. Finally, our conclusions are presented in “Conclusion” section.

## Materials and methods

There are two alternative beamline configurations available at Dingo—high-resolution and high-intensity, which use 1 cm and 2 cm diameter pinholes, respectively, and operate at different beam heights separated by approximately 20 cm. The high-resolution mode delivers a uniform field of $1.15 \times 10^{7}$ n cm$^{-2}$, while the latter provides a $4.7 \times 10^{7}$ n cm$^{-2}$ beam particularly suitable for phase contrast imaging^36^. Monte Carlo simulations were performed using Geant4 version 10.07 patch 2, which we chose in this work due to its open-source licensing, the ease with which the physics models in the code can be manually modified, and the extensive institutional expertise at ANSTO and the University of Wollongong with this toolkit^37–40^. Geant4 has previously been used for many similar projects, including neutron transport as well as the design of the neutron beam and beamline components^41–43^. For all simulations, inter-simulation variation was estimated for 95% confidence intervals. The physics models used in the simulations are listed in Table 1. Two circular 5 cm and 6 cm diameter beams, travelling in the negative *z* direction, were used to simulate both operation modes. The *L*/*D* ratios, where *L* is the distance between the entrance aperture of the beam to the image plane (detector box scintillator screen) and *D* is the diameter of the beam aperture^44^, are 1000 and 500 for the bottom and top pinhole, respectively. The distance between the collimator entry and the centre of the sample stage assembly in the model, where the in-beam scoring plane is located, is 990 cm. Maximum neutron acceptance angles were calculated for both modes. The angular $\sigma _x$ and $\sigma _y$ were set to 1.0$^\circ$ (which is increased from the geometric pinhole divergence of 0.34$^\circ$ to account for the perturbation along the beam transport system, i.e. interaction with, or shallow penetration through, the collimator or secondary shutter inner walls). To improve the simulation performance, the complete wall around the reactor core has not been modelled and any neutrons backscattered from the part of the primary shutter wall are killed as they will not cross the sample stage. The portion of these neutrons potentially rejoining the beam and actually being collimated towards the sample stage would be negligible due to their entry angles relative the to central beam axis leading to neutron absorption in $^{10}$B$_4$C coating on the in-pile collimator, primary shutter wall or other components downstream.

**Table 1:** Geant4 physics models used in the simulations.

Interaction	Energy range	Geant4 model
Radioactive decay	N/A	G4RadioactiveDecayPhysics
Particle decay	N/A	G4Decay
Hadron elastic	0–100 TeV	G4HadronElasticPhysicsHP
Ion elastic	0–110 MeV	Binary light ion cascade
	100 MeV–10 GeV	BIC
Neutron capture	0–20 MeV	NeutronHPCapture
	19.9 MeV - 100 TeV	nRadCapture
Neutron fission	0 eV–20 MeV	NeutronHPFission
	19.9 MeV–100 TeV	G4LFission
Neutron elastic	0 eV–20 MeV	NeutronHPElastic
	20 MeV–100 TeV	hElasticCHIPS
Neutron inelastic	0 eV–20 MeV	NeutronHPInelastic
	19.9 MeV–6 GeV	Binary cascade

### Simulation configuration

**Figure 1:**
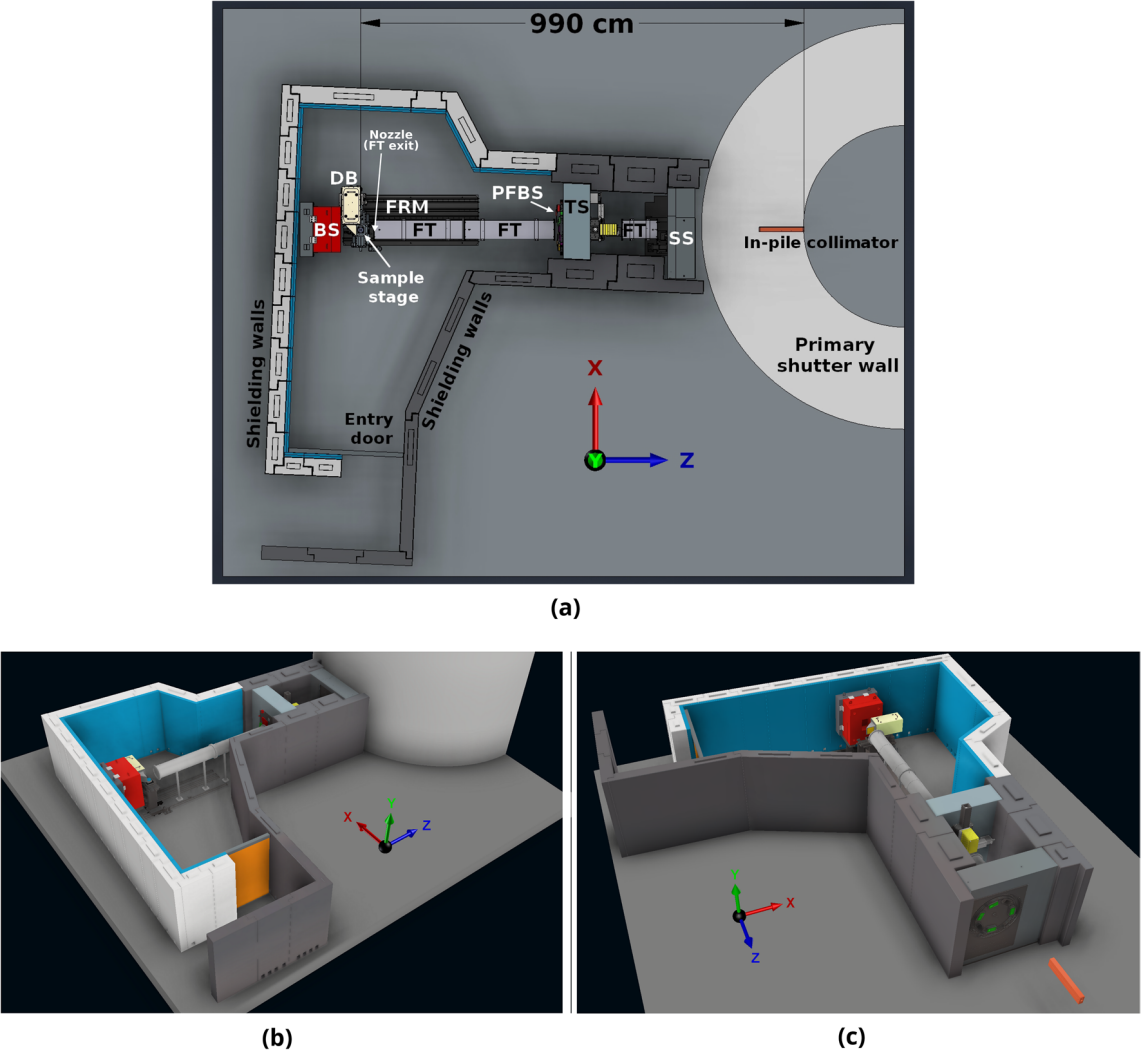
ACNS Dingo beamline model—(**a**) schematics of the beamline structure, where *SS* secondary shutter, *FT* flight tubes, *TS* tertiary shutter, *PFBS* pre-flight tube beam slits, *FRM* floor rail mounting, *DB* detector box, *BS* beam stop; (**b,c**) visualisation generated using Autodesk Inventor 2022. Shielding roof has been removed in the visualisation. The primary shutter wall has been additionally omitted in (**c**).

The Monte Carlo simulation model was constructed based on the CAD drawings of the beamline and information about the material compositions, including the in-pile collimator, beam transport system, hutch, sample room, instrumentation, beam stop and shielding assemblies and other components. The complete schematic of the ACNS Dingo beamline is depicted in Fig. 1a; views of the model visualised in Autodesk Inventor 2022 are presented in Fig. 1b,c. Each component model was constructed from its individual elements and preprocessed to ensure its correct position and orientation in space and checked for overlaps. Additional components and/or their elements were also modelled, including:

- Part of the primary shutter wall surrounding the reactor core.
- Standard concrete floor.
- Borated polyethylene lining on the shielding walls and roof in the sample room.
- Borated polyethylene entry door.
- $^{10}$B$_{4}$C coating on the in-pile collimator and secondary shutter inserts.
- Additional steel plates attached to the secondary shutter assembly.
- $^{10}$B$_{4}$C plates as part of the pre-flight tube slits.
- Helium gas cavity inside the flight tubes; and
- ZnS-$^6$LiF layer of the scintillation screen.

Fully closed structures exported in the Stereolithography (STL) format were imported into Geant4 using CADMesh interface, which translates the STL facets into G4TriangularFacets and creates G4TessellatedSolids^35,45^. Geant4 geometry tests with a femtometer tolerance threshold were carried out for each individual element to detect overlaps. These tests randomly sample points on the surface of a volume and determine whether they are potentially outside the volume and/or inside other volumes. The complete list of the beamline components included in the model, the composition of all materials defined in Geant4 and a summary of available methods to preprocess and import CAD drawings into Geant4, can be found in the Supplementary Materials.

### Neutron fluence experimental measurements with gold activation foils and $^{10}$B$_{4}$C-coated microdosimeter

**Figure 2:**
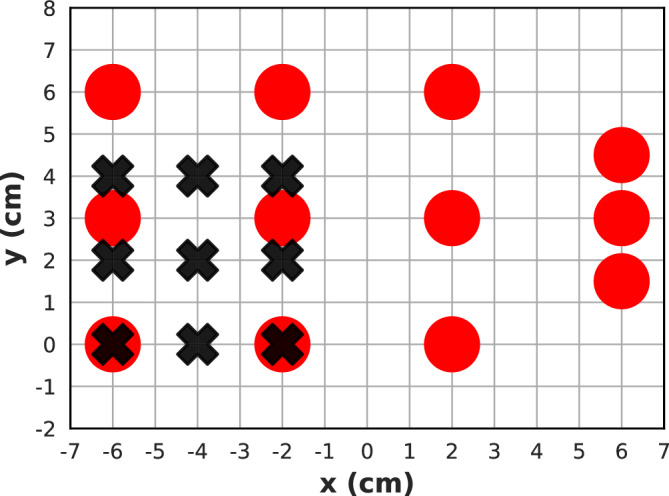
Schematic drawing of the experimental setup, where circles denote the gold foils, and **x** denotes $^{10}$B$_{4}$C-coated microdosimeter locations.

The thermal neutron fluence was measured using 1 cm diameter circular gold activation foils, manually attached to a well plate and aluminium holder and moved through 12 different positions along the beam *x* and *y* axes, with 4 cm and 1.5 cm steps in the *x* and *y* directions, respectively. Foils were exposed twice to a high-resolution mode 10 cm $\times$ 10 cm neutron field for 60 minutes, and the average of the two activity measurements for each location was used in the subsequent analysis^46–48^. Additionally, a microdosimeter covered with $^{10}$B$_{4}$C was placed in 9 locations using a motorised (*xy*-plane) sample stage within the 10 cm $\times$ 10 cm field^49^. Microdosimeter positions were stepped with a resolution of 2 cm along both *x* and *y* axes, and were irradiated for 15 min (see Fig. 2). The total energy deposition in the Sensitive Volume (SV) of the microdosimeter was used to measure the relative intensity of the thermal neutron fluence^34^. The results were normalised in two dimensions to the respective maximum experimental gold foil and microdosimeter readings.

### Simulation of the neutron field spatial distribution at the sample stage

In this work, a 40 cm $\times$ 40 cm scoring plane with a 0.8 mm$^2$ bin size was used to quantify the spatial fluence distribution of the 10 $\times$ 10 cm neutron beam at the sample stage and obtain precise information on the gradient across the field and in the penumbra region. The planar neutron distributions are plotted as 2D grayscale heatmaps, and line-profiles are generated along the *x*-axis (see Fig. 4). High-resolution and high-intensity fields were evaluated by plotting line-profiles at the centre of the field and at an offset of $+$ 5.5 cm in the *y*-direction, and by comparing 90% and 20% isocurves at the central *x*-axis. The high-resolution field was then compared to the experimental measurements with gold foils and $^{10}$B$_4$C-coated microdosimeters. The results were normalised in two dimensions to the maximum gold activation or $^{10}$B$_4$C-coated microdosimeter reading and the maximum of the simulated neutron field spatial distribution at that location. Finally, the percentage difference in neutron fluence between both datasets for each location was calculated, as per (1), by taking an average across a 1 cm fraction of the line-profile, which is equal to the gold foil diameter and 0.5 cm fraction for the microdosimeter results. The experimental gold activation foil and $^{10}$B$_4$C-coated microdosimeter measurements had a positioning accuracy of ± 0.5 cm and ± 0.25 cm.

1 $$\begin{aligned} \Delta \Phi =\frac{\Phi _{sim}-\Phi _{exp}}{\left[ \frac{\Phi _{sim}+\Phi _{exp}}{2}\right] } \times 100\%. \end{aligned}$$

**Figure 3:**
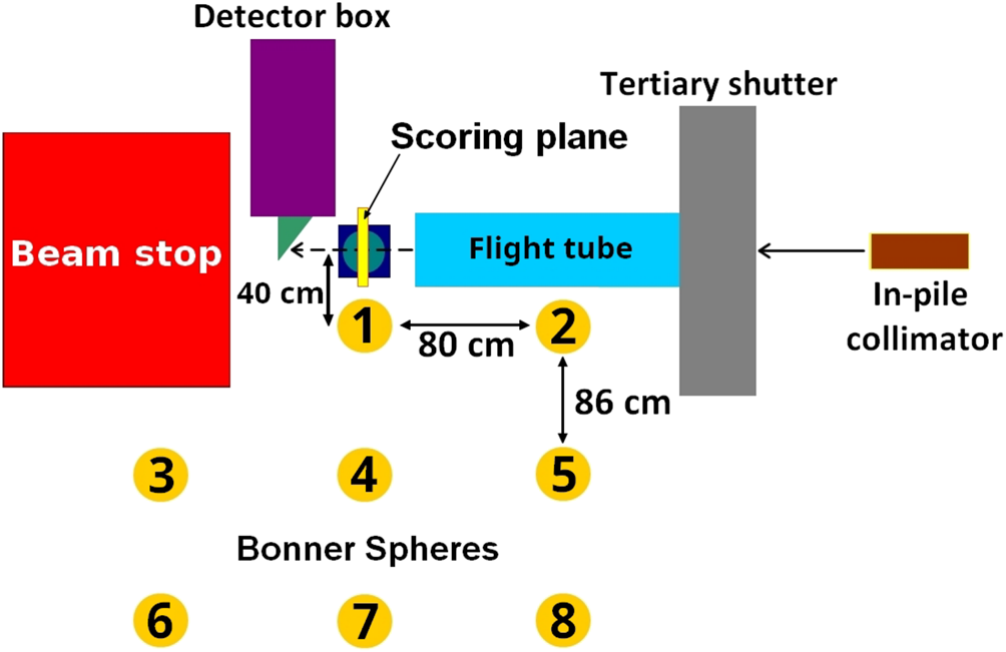
Simulation configuration illustrating the scoring plane used for the beam uniformity study and prediction of the in-beam neutron spectra, and the Bonner sphere experimental configuration.

**Figure 4:**
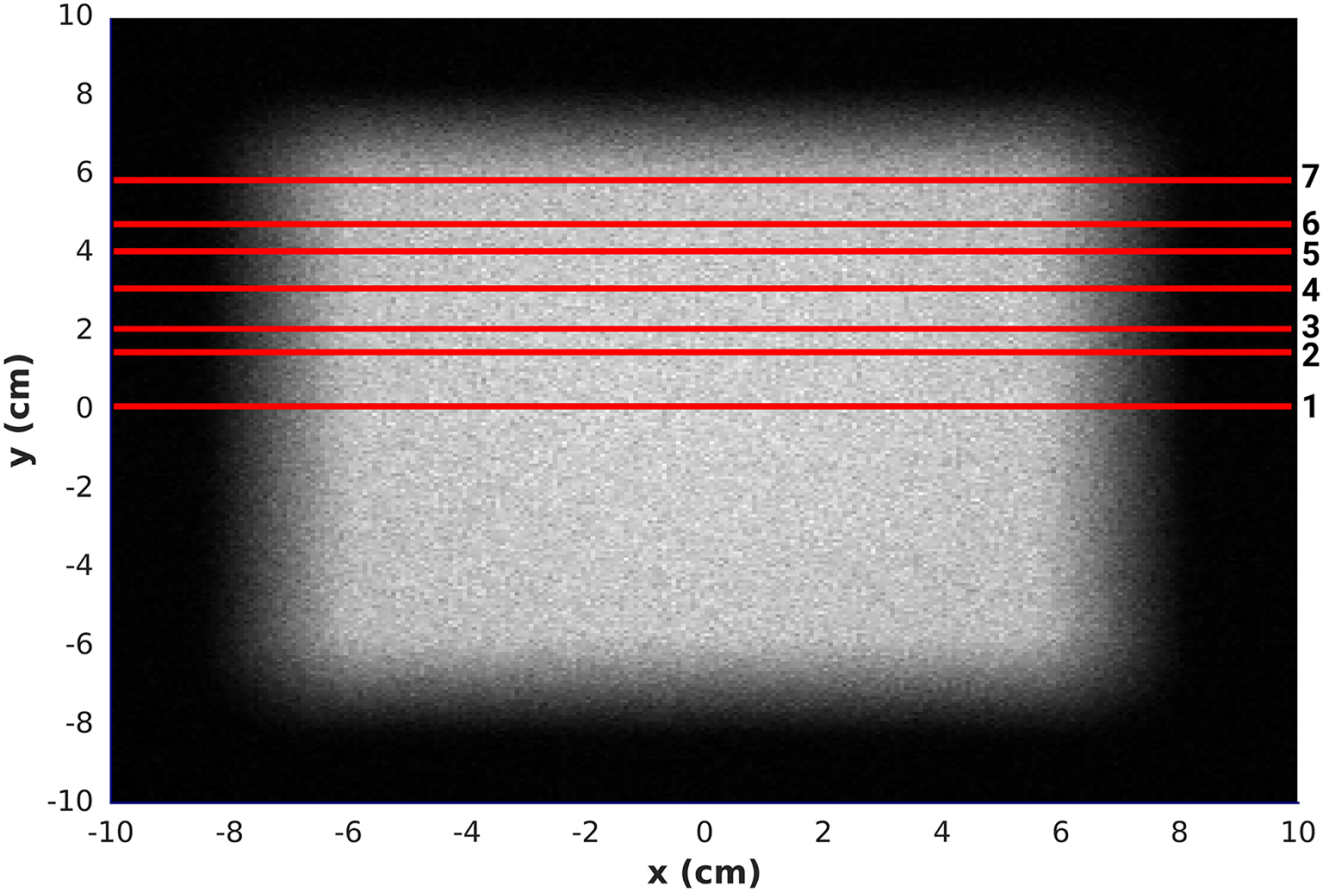
Locations of the horizontal line-profiles. The locations are numbered 1-7 from bottom to top.

### Absolute out-of-beam Bonner spheres spectroscopy

Neutron energy spectra were measured experimentally with a set of 2$^{\prime \prime }$, 3$^{\prime \prime }$, 5$^{\prime \prime }$, 8$^{\prime \prime }$, 10$^{\prime \prime }$ and 12$^{\prime \prime }$ Bonner spheres, as well as a bare detector and a detector covered with cadmium, placed at 8 different locations on the left-hand side of the nozzle, parallel to the central *z*-axis of the primary neutron beam (see Fig. 3). Each configuration was exposed to five-minute irradiation with a 20 cm $\times$ 20 cm high-intensity beam, with the experiment repeated three times. In this work, the neutron spectra were iteratively unfolded with the BUNKIUT code using SPUNIT algorithm. We used the MAXIET algorithm included in the BUNKIUT code to calculate the 1/*E* and Maxwellian initial guess spectrum. The parameters are listed in Table 2, where the 31 unequal intervals cover the neutron energy range up to 400 MeV. The 4 mm $\times$ 4 mm SANNA matrix represents a response to a 4 mm $\times$ 4 mm LiF detector^50,51^. The unfolded neutron spectrum at location 1 was used as a starting point (input spectrum) in the simulations.

**Table 2:** Parameters used to unfold the neutron spectra with BUNKIUT code^50,51^.

Parameter	Value
Number of energy intervals	31
Matrix type	4 mm $\times$ 4 mm SANNA
Unfolding algorithm	SPUNIT
Maxwellian temperature	2.8 MeV
Shape	0.0
Perturbation	0.0
End test	0.1
Smoothing factor	0.0
Calibration factor	1.0
Number of iterations before error test	10
Maximum number of iterations	1000

### Simulation of absolute out-of-beam neutron spectra

The out-of-beam neutron spectra were simulated by scoring neutron kinetic energies in the air at the same eight locations as during the experiment and were plotted as fluence per unit lethargy to improve the visualisation of the results by using logarithmically changing bins, where the neutron lethargy (*u*) is defined as follows^50^:

2 $$\begin{aligned} \Delta u = \log \left( \frac{E_{j+1}}{E_j}\right) . \end{aligned}$$

The predicted and unfolded neutron spectra, normalised to the reported high-intensity mode neutron fluence, were compared by calculating the percentage difference in the neutron fluence in the following energy windows: *A* (E$_{thermal}<$ 0.414 eV), *B* (0.414 eV < E$_{epithermal}<$ 11.7 keV) and *C* (E$_{fast}>$ 11.7 keV). The energy thresholds represent the bin edges generated by the BUNKIUT code, which were the nearest to the typical energy bands reported in the literature^52^. The input neutron spectrum in Geant4 was then iteratively adjusted by changing neutron fluence within individual bins until the maximum logarithmic ratio (simulation to experiment) in each bin was no higher than 2 and the ratio of neutron fluences was within 0.5 for each location and all three energy windows.

Finally, the input neutron spectrum in the model which achieved the best agreement with the unfolded experimental out-of-beam Bonner Sphere data was used to predict the in-beam neutron spectrum at the sample stage using a 20 cm $\times$ 20 cm high-intensity beam. The results were then compared to the predicted and measured out-of-beam spectra at location 1, and the contribution of the considered neutron components to the total neutron flux was calculated. To directly compare the results, the experimental and simulated out-of-beam neutron spectra were scaled using the average simulated in-beam to out-of-beam neutron fluence ratio.

The ratio of the simulated spectrum to the experimental spectrum was plotted on a logarithmic scale (i.e. $\log _{10}(\mathrm {sim/expt})$) to avoid under- or overrepresentation of the discrepancies between the simulation and experimental results and due to the dynamic range of the difference between individual bins. The logarithmic ratios of the average values obtained in each of the thermal, epithermal and fast regions of the spectra for the simulation and experimental measurements are also calculated and tabulated.

## Results

The model of the Dingo beamline comprises 14 major components listed in Table 3, which were divided into over 2400 individual elements (screws, jam nuts etc.).

**Table 3:** Major components of the ACNS Dingo beamline included in the model, where grades of the aluminium, steel and stainless steel alloys are 3003, ST52 and 304, respectively.

Component	Material composition
Part of the primary shutter wall	Standard concrete
Bunker shielding walls, roof, floor, entry door	Borated polyethylene, heavy concrete, standard concrete
In-pile collimator	B$_4$C, steel
Secondary shutter assembly, incl. beam selector wheel	Aluminium, stainless steel, steel
Secondary shutter inserts	Aluminium, stainless steel
Flight tubes	Aluminium, helium gas (1 atm), stainless steel, viton
Tertiary shutter assembly	Aluminium, boroflex, borated polyethylene, lead, polyethylene, stainless steel, standard concrete
Fast shutter assembly	Aluminium, polyethylene, steel
Pre-flight tube beam slits	Aluminium, B$_4$C
Floor rail mounting assembly	Aluminium, steel
Sample stage	Aluminium, stainless steel
Detector stage assembly (incl. CCD camera box)	Aluminium, lead, ZnS-$^6$LiF scintillator, stainless steel, steel
Beam stop assembly	B$_4$C, borated polyethylene, paraffin wax, polyethylene, stainless steel, steel

### Validation of the simulated neutron field spatial distribution

**Figure 5:**
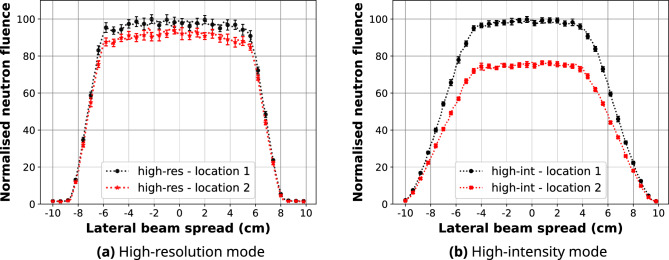
Line-profiles of the simulated 10 cm $\times$ 10 cm neutron fields at the sample stage position along the central axis (black) and + 5.5 cm away from the centre (red).

**Figure 6:**
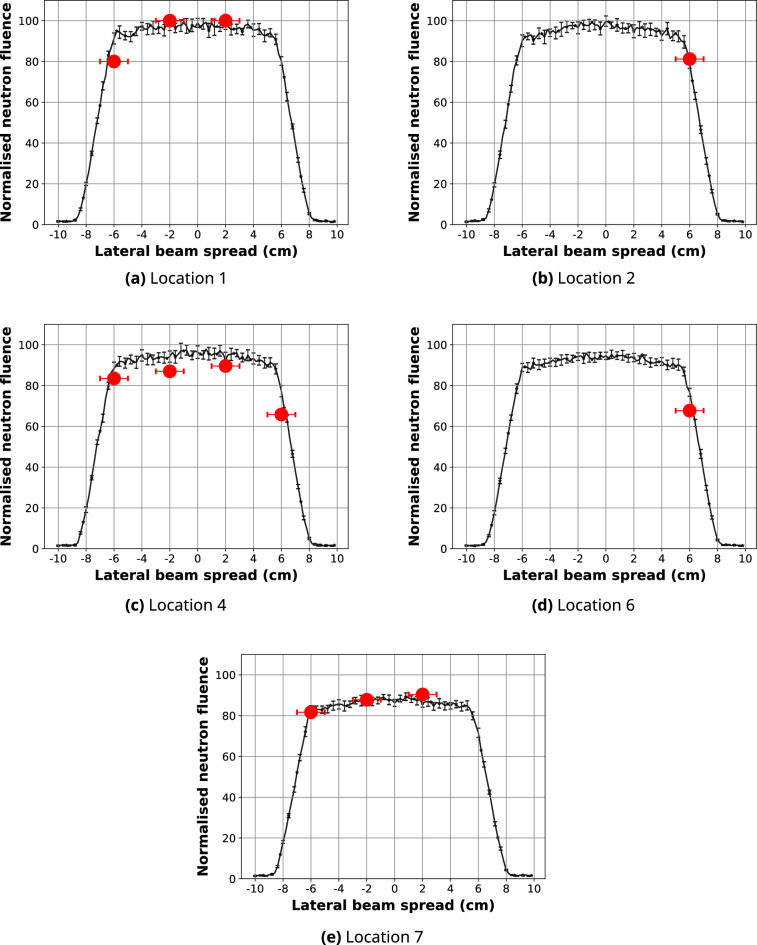
Line-profiles along the *x*-axis of the simulated 10 cm $\times$ 10 cm high-resolution neutron field after 2D normalisation to the maximum gold activation foil reading at Location 1 (black), compared to the experimental gold foil results (red circles). Positional variation in the experimental results is within ± 0.5 cm in *x* (error bars shown) and *y* directions. 95% confidence intervals for the simulated data are also shown.

**Figure 7:**
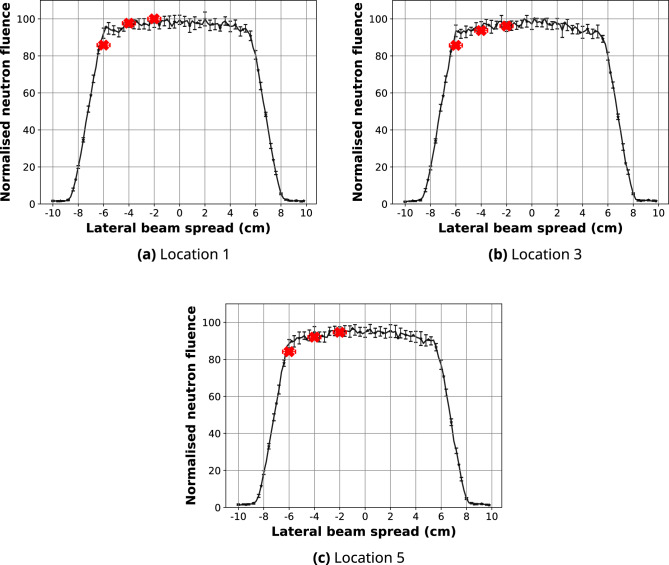
Line-profiles along the *x*-axis of the simulated 10 cm $\times$ 10 cm high-resolution neutron field after 2D normalisation to the maximum microdosimeter reading at Location 1 (black), compared to the experimental $^{10}$B$_{4}$C-coated microdosimeter results (red “**x**”). Positional variation in the experimental results is within ± 0.25 cm in *x* (error bars shown) and *y* directions. 95% confidence intervals for the simulated data are shown.

The predicted line-profiles of the 10 cm $\times$ 10 cm high-resolution and high-intensity neutron fields after 2D normalisation are presented in Fig. 5. The spatial distribution of the 10 cm $\times$ 10 cm high-resolution neutron field was compared to the experimental neutron fluence measurements with gold foils and $^{10}$B$_{4}$C-coated microdosimeters shown in Figs. 6 and 7, respectively. Calculated differences between the simulated and measured planar thermal/epithermal neutron distribution with both sets of detectors are listed in Tables 4 and 5.

**Table 4:** Percentage difference between the simulated and experimental gold activation foil neutron fluence.

Location	*x* position (cm)	$\Delta \Phi (\%)$	95% confidence interval
1	− 6 ± 0.5*	8.3	± 5.0
	− 2 ± 0.5	**− 2.9**	± 4.0
	2 ± 0.5	**− 2.4**	± 5.5
3	6 ± 0.5*	− 6.8	± 4.5
4	− 6 ± 0.5*	**1.7**	± 4.6
	− 2 ± 0.5	8.2	± 6.0
	2 ± 0.5	**5.5**	± 5.9
	6 ± 0.5*	13.0	± 4.5
6	6 ± 0.5*	8.3	± 3.5
7	− 6 ± 0.5*	− 5.7	± 3.6
	− 2 ± 0.5	**− 0.5**	± 4.0
	2 ± 0.5	**− 3.7**	± 3.9

The bold percentage differences show the locations where the agreement is within the inter-simulation variation. Locations in the penumbra region are indicated with*.

**Table 5:** Percentage difference between the simulated and experimental $^{10}$B$_{4}$C-coated microdosimeter neutron fluence.

Location	*x* position (cm)	$\Delta \Phi (\%)$	95% confidence interval
1	− 6 ± 0.3*	2.2	± 4.6
	− 4 ± 0.3	1.0	± 4.4
	− 2 ± 0.3	2.1	± 3.9
3	− 6 ± 0.3*	2.0	± 5.0
	− 4 ± 0.3	1.5	± 5.6
	− 2 ± 0.3	0.6	± 5.7
5	− 6 ± 0.3*	0.3	± 4.5
	− 4 ± 0.3	1.7	± 4.8
	− 2 ± 0.3	1.0	± 5.1

Locations in the penumbra region are indicated with*.

### Validation of the simulated out-of-beam neutron energy spectra

**Figure 8:**
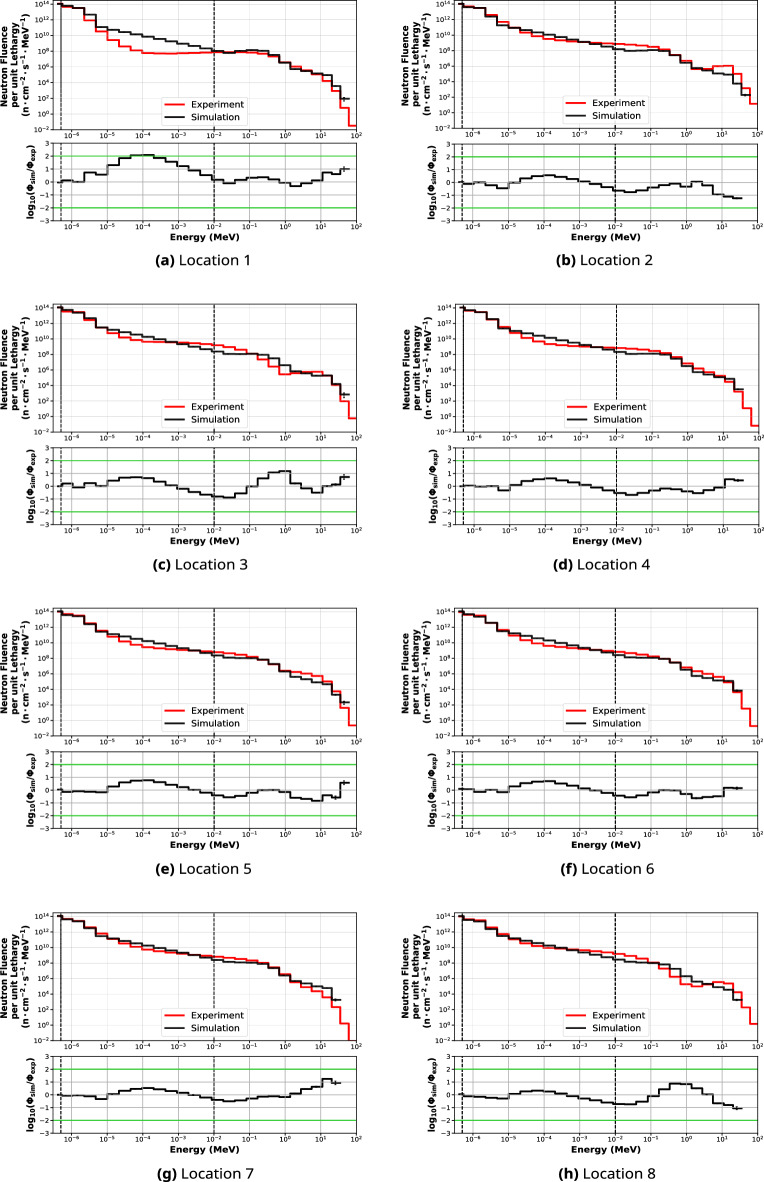
Normalised simulated (black) and experimental (red) out-of-beam neutron fluence per unit lethargy (upper graphs) and the percentage differences (lower graphs). 95% confidence intervals for the simulated data are shown.

**Figure 9:**
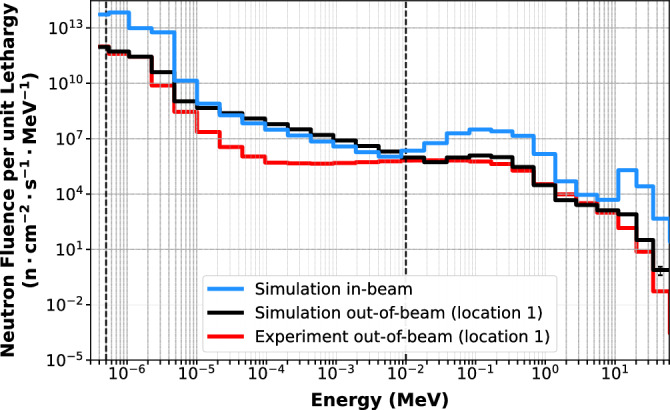
Neutron spectra simulated in-beam (blue) at the sample stage and out-of-beam (black) at location 1. Red line denotes the unfolded Bonner sphere neutron spectrum at location 1.

The normalised simulated and unfolded out-of-beam neutron spectra per unit lethargy and per bin energy are shown in Fig. 8. The comparison between simulated and experimental spectra is displayed for all neutron energy intervals, expressed as a ratio in Table 6. The energy threshold used for the calculation and the error bars for the unfolded spectra are given by the BUNKIUT code. The predicted in-beam and out-of-beam (Location 1) neutron energy spectra per unit lethargy are shown in Fig. 9, while Table 7 presents the calculated neutron fluences in each energy window. The results were normalised to the maximum gold activation foil reading, which corresponds to the thermal and epithermal neutron fluence, as well as the respective bin energy.

**Table 6:** The logarithmic ratios of average simulated to experimental fluences in the thermal (E$_{th}<$ 0.414 eV), epithermal (0.414 eV < E$_{epi}<$ 11.7 keV) and fast (E$_{fast}>$ 11.7 keV) spectral regions, for each of the 8 evaluated locations.

Location	log$_{10}$($\overline{\Phi _{sim}}/\overline{\Phi _{exp}}$)			
	Thermal	Epithermal	Fast	Overall
1	− 0.03 ± 0.001	0.15 ± 0.001	0.23 ± 0.002	0.13 ± 0.001
2	0.02 ± 0.002	− 0.08 ± 0.001	− 0.4 ± 0.003	− 0.19 ± 0.001
3	–	− 0.03 ± 0.004	− 0.03 ± 0.003	− 0.03 ± 0.002
4	–	− 0.01 ± 0.002	− 0.31 ± 0.003	− 0.12 ± 0.002
5	0.04 ± 0.000	− 0.07 ± 0.002	− 0.24 ± 0.004	− 0.10 ± 0.002
6	0.10 ± 0.000	− 0.02 ± 0.004	− 0.20 ± 0.004	− 0.06 ± 0.003
7	–	− 0.05 ± 0.004	− 0.23 ± 0.004	− 0.10 ± 0.003
8	0.04 ± 0.000	− 0.2 ± 0.004	− 0.15 ± 0.004	− 0.14 ± 0.003

The bin edges of the three neutron energy windows were selected based on the BUNKIUT code output. Results are normalised to the maximum neutron fluence measured with gold activation foils.

**Table 7:** Normalised predicted and experimentally measured neutron flux in each energy interval.

Location	$\Phi _{thermal}$	$\Phi _{epithermal}$	$\Phi _{fast}$
Simulated (sample stage)	1.03 $\times$ 10$^{7}$	3.67 $\times$ 10$^{7}$	1.01 $\times$ 10$^{7}$
Simulated (out-of-beam, location 1)	1.15 $\times$ 10$^{5}$	2.94 $\times$ 10$^{5}$	1.86 $\times$ 10$^{5}$
Measured (out-of-beam, location 1)	2.46 $\times$ 10$^{5}$	1.63 $\times$ 10$^{5}$	2.38 $\times$ 10$^{5}$

## Discussion

The model of the ACNS Dingo beamline has been imported into Geant4 and passed repeated Geant4 overlap tests. The simulation results show that for the high-resolution mode 10 cm $\times$ 10 cm beam, the 90% and 20% isocurves cover areas of approximately 10.5 cm $\times$ 10.5 cm and 15.75 cm$~\times$ 15.75 cm, respectively. For the high-intensity mode, these isocurves correspond to areas of around 8.5 cm $\times$ 8.5 cm and 16.2 cm $\times$ 16.2 cm. The intensity at a distance 5.5 cm away from the centre drops by 5–10% and 20% when using the high-resolution and high-intensity modes, respectively. The latter provides roughly 50% higher fluence at the centre of the field.

The percentage difference between the simulated planar thermal/epithermal neutron distribution and measured neutron fluence was calculated for each experimental location. Some of the data points—specifically, those in locations 1, 3, 4, 6 and 7—fall into the penumbra region; in these regions, the maximum percentage difference of 12.9% was observed. Neutron fluence at the centre of the field is within 8.1% of the gold activation measurement and within 2.1% of the microdosimeter data. In most cases, $\Delta \Phi$ is below 3.7% and 1.5% for the gold foil and microdosimeter measurements, respectively. We found a higher agreement between the predicted planar neutron distribution obtained with a microdosimeter compared to the gold foil measurements. This is likely due to the better positional accuracy which can be obtained with the microdosimeter, which is especially noticeable in the penumbra region; the microdosimeters were positioned using a motorised *xy*-translation table, while gold foils were manually attached to a well plate and an aluminium holder, leading to some positioning error. The magnitude of the error bars in the simulated line-profiles is due to the resolution of the scoring plane.

Characterisation of the neutron spectrum is essential to quantify the direct and indirect impact of the neutron beam on biological media. Nuclear reactions within a biological target can lead to the production of secondary high linear energy transfer (LET) charged particles. The magnitude of this effect, and therefore the relative biological effectiveness (RBE), depends on the neutron energy and the reaction channels being open^53,54^. Moreover, fast neutrons may cause damage to the instrumentation and historical samples.

The logarithm of the simulated and measured total neutron fluence ratio is within the range ± 0.2. For epithermal neutron fluence, the largest log-ratios of approximately 0.15 and − 0.2 are observed at locations 1 and 8, respectively. For all other locations, the log of the simulated to measured fluence ratio is below − 0.08. The fast neutron fluence log-ratio is less than or equal to ± 0.4 across all locations, mainly because of single bin discrepancies at the peak around 10–100 keV. The larger deviations between simulation and experimental measurements observed in some locations may be due to several reasons, including model inaccuracies, objects present in the physical room but not yet included in the simulation model, differences between the idealised materials used in the simulation model and the actual materials used for the construction of the beamline, the accuracy of the unfolding process, detector positional accuracy, or variation in reactor performance, which is not taken into account in the analysis. In fact, the BUNKIUT code produces an interpolated probability density distribution, while the raw simulation results are expressed as counts per energy bin. This may be especially apparent in the fast neutron range above 10 MeV, where a single neutron in the simulation may cause a difference of several orders of magnitude when compared to the probability below the value of 1 given by the unfolding code.

The epithermal component of the beam was predicted to constitute approximately 49% and 64% out-of-beam and in-beam, respectively. Fast neutrons do not scatter as widely as thermal and epithermal neutrons, however, it was found that the predicted fast neutron component out of the field and at the sample stage was around 32% and 18% of total neutron fluence, which is significant from a radiobiological point of view. The predicted spectra exhibit a distribution pattern typical for thermal reactors, and consist of a thermal Maxwellian, followed by a flat epithermal neutron spectrum and a fission-like fast neutron component^55^. It is also similar to spectra reported for other thermal neutron beamlines, such as the Neutron Radiography Reactor (NRAD) at the Idaho National Laboratory (INL) in USA^56^, Thermal and Epi-thermal Neutron Irradiation Station (TENIS) at the Institut Laue-Langevin in France^57^, Syrian Miniature Neutron Source Reactor (MNSR)^58^, or Kalpakkam Mini reactor (KAIMINI) in India^59^. Giegel et al.^56^ reported that the epithermal and fast neutron components are approximately 2 and 10 times higher than the thermal component. This ratio appears to be around 4 and 1 for the Dingo beamline suggesting a different shape of the neutron energy distributions, particularly in the fast neutron region. However, distinct neutron energy windows were used in both studies so that a direct comparison is not possible.

Due to the flexibility and cost-effectiveness of Monte Carlo simulation methods, our developed model provides convenient means to estimate the neutron spectra at any location within the beam transportation system, which will be useful for designing, planning and evaluating upgrades or modifications to the beamline prior to physical implementation and/or installation. These could include modifications to the neutron spectrum exiting the nozzle, i.e. through the design of neutron filters for selecting specific neutron energy bands. It may be of particular significance for planning biological experiments or evaluating prototype instrumentation for NCEPT or NCT, where thermal and epithermal energy windows are desired. Moreover, the simulation model can provide detailed information on phenomena that are challenging to be measured experimentally, such as the production of short-lived and trace isotopes or prompt gamma emission. Finally, the existence of such a model allows simulating experimental design and optimising its configuration.

## Conclusion

In this work, a validated Monte Carlo model of the ACNS Dingo beamline has been developed and used to predict the in-beam neutron spectra at the sample stage position. The model can either be used directly or the neutron field that it generates can be provided to users in the form of a phase-space file, reducing the computational workload. Spatial characteristics of the simulated beam have been validated against experimental neutron fluence measurements with activated gold foils and a $^{10}$B$_{4}$C-coated SOI microdosimeter, and were found to be within 8.1% and 2.1% at the centre of the field, respectively. The average logarithmic neutron fluence ratios between the out-of-beam Bonner sphere measurements and simulation results in the thermal (E$_{th}<$ 0.414 eV), epithermal (0.414 eV < E$_{epi}<$ 11.7 keV) and fast (E$_{fast}>$ 11.7 keV) neutron regions were within ± 0.1, ± 0.2 and ± 0.4. The epithermal and fast neutron components out-of-beam were found to be approximately 49% and 31% of total neutron fluence. The predicted neutron spectrum at the sample stage position consists of approximately 18% thermal, 64% epithermal and 18% fast neutrons. The next stage of this work will include a comparison of simulation and experimental gamma spectra in the out-of-beam region, which will be used to indirectly validate the in-beam gamma spectrum.

### Supplementary Information

Supplementary Information.

## Data Availability

All data generated or analysed during this study are included in this published article (and its Supplementary Information files) or are available from the corresponding author on reasonable request.
