# Supplementary material for: A Monte Carlo model of the Dingo thermal neutron imaging beamline
Source: Sci Rep. 2023 Oct 13;13:17415. doi: 10.1038/s41598-023-44035-4 (PMC10575880; doi:10.1038/s41598-023-44035-4)
Supplement: Supplementary file 1 — Supplementary Information. [file 41598_2023_44035_MOESM1_ESM.pdf]

1 Supplementary materials

1.1 Review of the methods to import CAD files into Geant4

Computer-Aided Design (CAD) drawings in a Standard for the Exchange of Product Data (STEP) format cannot be directly imported to Geant4. To load the geometry, STEP files have to be converted to a format recognisable by Geant4, such as Geometry Description Markup Language (GDML) or Standard Triangle Language (STL) used for Stereolithography. Geant4 has an integrated GDML parser that uses Xerces-C++ software packages to import and export GDML files[1]. There are tools available to convert STEP to GDML, for example, FastRad, ESABASE2 or SW2GDML, which converts SolidWorks CAD files to GDML[2]. GUIMesh allows to import STEP files using open-source FreeCAD libraries and python Graphical User Interface, tessellate the volumes and export to GDML[3]. STEP files can also be converted to STL using FreeCAD, SolidWorks, AutoCAD or Autodesk Inventor. The output STL files (or alternative mesh formats, i.e. OBJ and PLY) can be then exported as a single assembly or individual elements and imported into Geant4 by using mesh2gdml or CADMesh[4,5]. The latter is a single header interface and does not require compilation or installation. Manipulation of the geometry in STL format is challenging since they are represented by triangular meshes. Nonetheless, if necessary, STL files can be converted back to STEP format using Autodesk's Meshmixer.

1.2 Dingo beamline Monte Carlo model

The Monte Carlo model of the Dingo beamline was preprocessed in Autodesk Inventor 2022 and consists of 14 major components as follows:

- Part of the primary shutter wall surrounding the reactor core;
- Standard concrete floor;
- Bunker shielding walls and roof;
- In-pile collimator;
- Secondary shutter assembly;
- 4 secondary shutter inserts (beam size selection);
- Tertiary shutter assembly;
- Fast neutron shutter assembly;
- 3 flight tubes varying in size;
- Pre-flight tube B_4C beam slits;
- Detector box with a CCD camera;
- Sample stage;
- Floor rail mounting assembly (for the detector box stage and sample stage);
- Beam stop;

These components comprise over 2400 individual STL files, which are grouped by component and material type, and are loaded into Geant4 via CADMesh. Tesselated solids are then assigned with relevant materials defined in Geant4; their compositions are presented in Table 1.

Table 1. Elemental composition of the materials used in the simulations. Percentage ratio rounded to two decimals.

Component	Density (g/cm^3)	Elemental composition
Al_2O_3	3.95	52.895% Al; 47.105% O
Aluminium alloy (3003 grade)[6]	2.79	97.5% Al; 1.0% Mn; 0.7% Fe; 0.6% Si; 0.1% Cu; 0.1% Zn
B_4C	2.45	75.9% ^{10}B; 2.4% ^{11}B; 17.0% C; 3.8% Mg; 0.6% O; 0.3% H
$BaSO_4$	4.5	58.85% Ba; 27.24% O; 13.74$ S
Borated polyethylene	1.0115	95% polyethylene; 5% ^{10}B
Boroflex	2.125	50% B_4C; 25% SiO_2; 25% polydimethylsiloxane (PDMS)
CaO	3.34	71.47% Ca; 28.53% O
CaF_2	3.18	51.33% Ca; 48.67% F
Fe_2O_3	5.24	69.94$ O; 30.06% Fe
FeO	5.74	77.73% Fe; 22.27% O
Heavy concrete[7]	3.67	45.95% magnetite; 23% sand; 15% Portland cement; 5.25% water; 1.8% silica fume
Helium gas (1 atm, 293 K)	0.000178	N/A
K_2O	2.35	83.01% K; 16.99% O
^{6}LiF (enriched)	2.635	95% ^{6}Li; 5% F
Magnetite	4.85	92.8% FeO; 3.3% CaO; 3% SiO_2; 0.4% Al_2O_3; 0.3% Na_2O; 0.2% K_2O
MgO	3.58	60.31% Mg; 39.69% O
N_2O	1.22	63.65% N; 36.35% O
Na_2O	2.27	74.185% Na; 25.815% O
Paraffin wax	0.9	85.02% C; 14.98% H
Polydimethylsiloxane (PDMS)	0.965	37.88% Si; 32.39% C; 21.58% O; 8.15% H
Polyethylene $(C_2H_4)_N$	0.94	86% C; 14% H
Portland cement[8]	3.28	63.1% CaO; 21.9% SiO_2; 6.9% Al_2O_3; 3.9% Fe_2O_3; 2.5% MgO; 1.7% SO_3
Sand[7]	2.66	94.84% SiO_2; 2.12% Al_2O_3; 1.23% Fe_2O_3; 0.69% K_2O; 0.52% CaO; 0.1% MgO; 0.27% Na_2O; 0.12% TiO_2; 0.11% SO_3
Silica fume[9]	2.62	97.25% SiO_2; 0.15% SO_3; 0.5% K_2O; 0.5% MgO; 0.5% Fe_2O_3; 0.5% water; 0.2% Al_2O_3; 0.2% N_2O
SiO_2	2.65	53.25% Si; 46.75% O
SO_3	1.92	59.95% O; 40.05% S
Stainless steel (austenitic, grade 304)	7.9	72.95% Fe; 18.5% Cr; 8.5% Ni; 0.05% C
Standard concrete	2.3	52.9% O; 33.7% Si; 4.4% Ca; 3.38% Al; 1.6% Na; 1.4% Fe; 1.3% K; 1% H; 0.2% Mg; 0.1% C
Steel (ST52)[10]	7.85	97% Fe; 1.6% Mn; 0.55% Si; 0.55% Cu; 0.22% C; 0.03% P; 0.03% S; 0.012% N
TiO_2	4.23	59.93% O; 40.07% Ti
ZnS-^{6}LiF scintillator	2.79	75% ZnS; 25% ^{6}LiF
ZnS	4.09	67.1% Zn; 32.9% S
Viton	99.2859	59% C; 41% F

References

1. Allison, J. *et al.* Recent developments in Geant4. *Nucl. Instruments Methods Phys. Res. Sect. A: Accel. Spectrometers, Detect. Assoc. Equip.* **835**, 186–225, DOI: 10.1016/j.nima.2016.06.125 (2016).
2. Vuosalo, C., Carlsmith, D., Dasu, S. & and, K. P. A tool to convert CAD models for importation into Geant4. *J. Physics: Conf. Ser.* **898**, 042024, DOI: 10.1088/1742-6596/898/4/042024 (2017).
3. Pinto, M. & Gonçalves, P. GUIMesh: A tool to import STEP geometries into Geant4 via GDML. *Comput. Phys. Commun.* **239**, 150–156, DOI: 10.1016/j.cpc.2019.01.024 (2019).
4. Graf, N. A. mesh2gdml. *J. Physics: Conf. Ser.* **396**, 022018, DOI: 10.1088/1742-6596/396/2/022018 (2012).
5. Poole, C. M., Cornelius, I., Trapp, J. V. & Langton, C. M. A CAD interface for GEANT4. *Australas. Phys. Eng. Sci. Medicine* **35**, 329–334, DOI: 10.1007/s13246-012-0159-8 (2012).
6. steels, A. Atlas steels. https://www.atlassteels.com.au. Accessed: 2022.08.01.
7. Ouda, A. S. Development of high-performance heavy density concrete using different aggregates for gamma-ray shielding. *Prog. Nucl. Energy* **79**, 48–55, DOI: 10.1016/j.pnucene.2014.11.009 (2015).
8. Dunuweera, S. P. & Rajapakse, R. M. G. Cement Types, Composition, Uses and Advantages of Nanocement, Environmental Impact on Cement Production, and Possible Solutions. *Adv. Mater. Sci. Eng.* **2018**, 1–11, DOI: 10.1155/2018/4158682 (2018).
9. Mohamed, H. A. Effect of fly ash and silica fume on compressive strength of self-compacting concrete under different curing conditions. *Ain Shams Eng. J.* **2**, 79–86, DOI: 10.1016/j.asej.2011.06.001 (2011).
10. Rextonsteel. Rexton steel and alloys. https://www.rextonsteel.com. Accessed: 2022.08.01.
